# Usefulness of School Absenteeism Data for Predicting Influenza Outbreaks, United States

**DOI:** 10.3201/eid1808.111538

**Published:** 2012-08

**Authors:** Joseph R. Egger, Anne G. Hoen, John S. Brownstein, David L. Buckeridge, Donald R. Olson, Kevin J. Konty

**Affiliations:** New York City Department of Health and Mental Hygiene, New York, New York, USA (J.R. Egger, K.J. Konty);; Boston Children’s Hospital, Boston, Massachusetts, USA (A.G. Hoen, J.S. Brownstein);; Harvard Medical School, Boston (A.G. Hoen, J.S. Brownstein);; McGill University, Montreal, Quebec, Canada (D.L. Buckeridge);; and International Society for Disease Surveillance, New York (D.R. Olson)

**Keywords:** influenza, viruses, school health, population surveillance, outbreaks, public health, prevention and control, pandemic, methods evaluation, New York, United States, absenteeism

**To the Editor:** School closure has been proposed as a strategy for slowing transmission of pandemic influenza (*1*). Studies of influenza A(H1N1)pdm 2009 (pH1N1) suggested that early and sustained school closure might effectively reduce communitywide influenza transmission (*2**,**3*). However, empirical evidence identifying the optimal timing of school closures to effectively reduce disease transmission after an outbreak occurs is limited.

That school absenteeism data improve school-based disease surveillance and response has been suggested (*4**–**6*). In 2009, Sasaki et al. demonstrated that the pattern of influenza-associated school absenteeism in the days before an influenza outbreak predicted the outbreak course with high sensitivity and specificity (*7*). However, that study used absenteeism data from Japan, which are generally not applicable to the United States, because most US absenteeism data collected at the local level do not specify cause. Furthermore, few US jurisdictions collect electronic health data for students.

In New York City (NYC; New York, New York, USA), electronic health data are collected daily on ≈70%–80% of the total nurse visits in the city’s public schools, kindergarten through grade 8, and on all-cause school absenteeism. Using these data, we adapted the algorithm developed by Sasaki et al. for use with all-cause absenteeism data from NYC schools and validated our findings by using the daily count of school nurse visits for fever/influenza over the same period (*7*). To reduce variance, we aggregated absenteeism data for September 6, 2005, through June 26, 2009, for 1,206 public schools in NYC at the school day and school district levels. A negative binomial regression model was then fit to these data, adjusting for day of week, whether the preceding day was a holiday, school type (elementary, middle), school day (linear term), and sine and cosine terms to account for seasonality. This modeling approach was used to standardize the outcomes across school districts and to further reduce variance caused by factors unassociated with influenza transmission.

A similar regression model was also fit to the daily school district–level count of school nurse visits for fever/influenza syndrome over the same period. However, for this model, seasonal influenza periods, determined by virus isolate data, were censored before modeling.

Residuals of both models were then used to calculate school district–specific z-scores for each day from September 25, 2006, through June 26, 2009, by dividing the model residual by the school district–specific standard deviation of the outcome. To determine the threshold and pattern in lagged days that best predicts an outbreak of absenteeism and fever/influenza syndrome, we applied the Sasaki et al. algorithm to the absenteeism z-score time series. We calculated receiver operating characteristic (ROC) curves by observing whether z-score thresholds of 1, 1.5, 2, or 2.5 reached either 1, 2, or 3 days in a row, were followed by an influenza outbreak in the same school district in the next 7 days. An influenza outbreak was indicated by a z-score of at least 3 (Technical Appendix).

Results revealed a moderately positive in-phase correlation between absenteeism and fever/influenza syndrome by school district during the pH1N1 period (r = 0.264) but a weak correlation over the entire study period (September 6, 2005–June 26, 2009) (r = 0.086). When data were aggregated across the city, the correlation between absenteeism and fever/influenza z-scores during the pH1N1 period and the entire study period increased to 0.304 and 0.210, respectively. When estimating a cross-correlation function to the citywide data, the absenteeism time series correlated most strongly with the fever/influenza syndrome time series at a 2-day lag (pH1N1 period, r = 0.550; entire study period, r = 0.213), indicating that changes in absenteeism were most strongly correlated with changes in fever/influenza syndrome visits 2 days earlier.

The ROC curves illustrate the limited ability of absenteeism and fever/influenza visit patterns to predict absenteeism and fever/influenza outbreaks (Figure). The ROC curves also show that absenteeism in the week before an outbreak has little ability to predict an outbreak of either fever/influenza syndrome or absenteeism during the entire study period or during a period of pandemic influenza.

**Figure:**
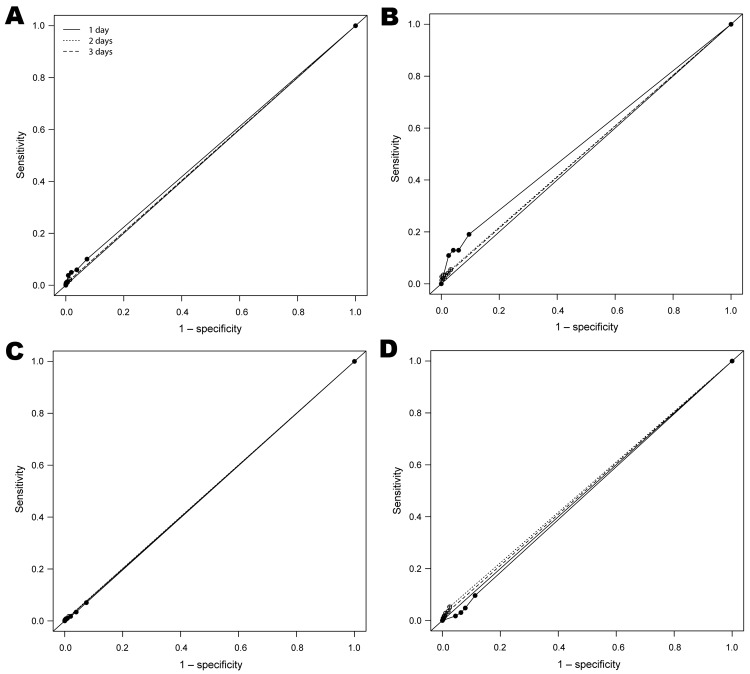
Receiver operating characteristic (ROC) curves showing A) predictive ability of school absenteeism to detect an outbreak (z-score ≥3) of fever/influenza for the entire study period; B) fever/influenza for the pandemic (H1N1) 2009 period; C) absenteeism for the entire study period; and D) absenteeism for the pandemic (H1N1) 2009 period. ROC curves were based on observations of whether 4 absentee threshold z-score levels—1, 1.5, 2, or 2.5—were reached or exceeded for either 1 day, 2 consecutive days, or 3 consecutive days as a predictor of the school district outbreak status during the next 7 days. In the ROC curves, sensitivity on the y-axis indicates the true-positive rate, and 1– specificity on the x-axis indicates the false-positive rate. The study was conducted September 6, 2005–June 26, 2009, in New York City, New York, USA.

Thus, non–disease-specific absenteeism data alone are of little use for school-based influenza surveillance. Use of all-cause absenteeism data cannot inform influenza mitigation policies, such as school dismissal, at the school or the school district levels. Not surprisingly, the influenza-specific absenteeism data from Japan were better able to predict an influenza outbreak than were our data because our data were not influenza specific. Other factors specific to the school system in Japan might have also played a role.

In the future, it might be beneficial for schools to collect causes of absenteeism, particularly if is it not feasible to electronically collect data on school nurse visits. Creation of school-based early warning systems for pandemic influenza remains a priority. In NYC, efforts to improve emergency department and primary care electronic medical record systems have been successful (*8**–**10*). Similar efforts to improve electronic health data collection and influenza-related absenteeism data in schools might yet demonstrate the usefulness of school-based surveillance systems.

Technical Appendix Statistical model development.
