# Supplementary material for: Usefulness of School Absenteeism Data for Predicting Influenza Outbreaks, United States
Source: Emerg Infect Dis. 2012 Aug;18(8):1375–7. doi: 10.3201/eid1808.111538 (PMC3414019; doi:10.3201/eid1808.111538)
Supplement: Technical Appendix — Statistical model development. [file 11-1538_Techapp-s1.pdf]

Article DOI: http://dx.doi.org/10.3201/eid1808.111538

Usefulness of School Absenteeism Data for Predicting Influenza Outbreaks, United States

Technical Appendix

Statistical Model Development

Daily counts of absenteeism and fever/influenza syndrome cases for 1,206 public schools in New York City were aggregated by school day and school district for the period September 6th, 2005 to June 26th 2009. This aggregation was performed to reduce the variance in both outcomes. The raw count data varied widely for absenteeism (mean percent absent = 12.7%, SD = 9.3%) and fever/influenza cases (mean count = 0.20, SD = 0.26) by school, by day. As a result, preliminary modeling of the raw case counts produced large residuals.

Separate negative binomial regression models were fit to the daily count of school absenteeism and a daily count of fever/influenza syndrome cases for the period September 6th, 2005 to June 26th, 2009, by school district. For both models, we used an offset term for the total school district enrollment for each day to adjust each count by school population (i.e., larger schools should have a larger number of fever/influenza cases). For the fever/influenza model, daily fever/influenza cases during influenza periods were removed before modeling. At the New York City Department of Health and Mental Hygiene, the start of a influenza period is defined as 2 consecutive weeks of 2 or more influenza isolates confirmed by a World Health Organization collaborating laboratory , and the end of a influenza period is the last week reporting $\geq$1 influenza isolates. Complete censoring of influenza periods from the data enabled us to estimate the expected excess count of fever/influenza cases in these schools. If we had not censored this period, we would have observed an ‘outbreak’ in the data only if the observed count was historically unusual, instead of seasonally unusual, which was the threshold we were seeking.

For both models, terms for day of the week, school type (elementary, middle school), and whether the preceding or proceeding day was a holiday were fit to the data because they were

believed to be independently associated with that day's absentee and fever/influenza syndrome counts. Linear, sine and cosine terms for the school day were also included in these models to account for linear and nonlinear changes in absenteeism and counts of fever/influenza syndrome that were observed upon visual inspection of the data. Inclusion of these covariates in the models to predict counts of absenteeism and fever/influenza helped to further reduce the variance in the data due to factors thought to be unrelated to ininfluenzaenza transmission. Both models resulted in adjusted, predicted counts of all-cause absenteeism and fever/influenza syndrome by school district. A z-score was then calculated by dividing the model residual (observed minus expected) by the school district-specific standard deviation of the outcome for each school district, for each day during the study period. Producing a model-estimated z-score of each outcome enabled us to remove some of the residual variance in the data as well as produce a normalized distribution of the outcomes for each school district to better determine an unexpected rise (i.e., an 'outbreak) for each outcome.
